# *baal-nf* identifies motif-disrupting variants that decrease transcription factor binding affinity

**DOI:** 10.1186/s13059-025-03916-9

**Published:** 2026-01-13

**Authors:** Breeshey Roskams-Hieter, Øyvind Almelid, Chris P. Ponting

**Affiliations:** 1 Institute of Genetics and Cancer, MRC Human Genetics Unit, Western General Hospital, University of Edinburgh, Edinburgh, EH4 2XU UK; 2 Health Data Research UK, 215 Euston Road, London, NW1 2BE UK

**Keywords:** ChIP-sequencing, Allele-specific binding, Allelic imbalance, Transcription factors, Motifs

## Abstract

Human traits vary in part due to genetically-determined change of transcription factor binding affinity within gene regulatory regions. However, few trait-causal variants or mechanisms are known. Here we propose 1,935 variants as strong candidates for causally altering human traits. We discover these through *baal-nf* which uses chromatin immunoprecipitation-sequencing data to identify allelic imbalance at heterozygous sites for affinity-concordant positions within transcription factor- and co-factor binding motifs. These allele-specific binding sites are evolutionarily conserved and enriched for trait and gene expression associations. *baal-nf* and these high-quality allele-specific binding sites allow trait variation due to altered transcription factor binding to be investigated.

**Supplementary Information:**

The online version contains supplementary material available at 10.1186/s13059-025-03916-9.

## Background

Revealing the molecular mechanisms by which DNA variants alter function is critical for understanding how genotypes contribute to physiological traits [1]. DNA variants associated with complex traits and disease mostly lie within non-coding regions, suggesting that trait variation is often caused by genetically altered transcription factor (TF) binding affinity [2–7]. Attributing trait variation to a particular TF and its altered site-specific binding affinity, however, remains an unsolved problem in population genetics and functional genomics. Its solution will require accurate and comprehensive catalogues of both TF binding motifs and affinity-altering DNA variants mapped to these motifs [8].

DNA variants that significantly alter TF binding have been inferred from chromatin immunoprecipitation with sequencing (ChIP-seq) studies. Rather than comparing across population samples, these experiments use the known genotypes of single cell lines to minimise false variant calls [9–12]. TF binding that favours one allele over the other (i.e., allele-specific TF binding sites; ASBs) is inferred at the cell line’s known heterozygous sites when there is a statistically significant imbalance of ChIP-seq reads mapped to one of the two alleles.

To reliably distinguish true allele-specific binding from artifacts, models that infer ASBs must account for biases, such as copy number variants (CNVs), especially in immortalised cancer cell lines carrying chromosomal rearrangements which create allelic imbalance in the underlying genomic DNA [9, 13]. Technical biases include: (i) reference mapping bias, caused by a systematic preference of mapping reads to the REF over the ALT allele; (ii) polymerase chain reaction (PCR) amplification bias, when reads carrying one of the two alleles are preferentially amplified during PCR; (iii) sequencing errors introduced during variant calling, when heterozygous SNPs or systematic base-calling errors systematically favour one allele; (iv) inaccurate read mapping often in low-complexity regions of the genome; and, (v) cellular heterogeneity, whereby ASB calls are confounded by non-uniform sample composition resulting in mixed ASB signals.

Although many non-motif-based features modulate TF binding affinity [8, 14, 15], TF binding motifs provide insight into molecular mechanism when an ASB maps to a well-defined motif for the experimentally-targeted TF, and when its lower affinity allele disrupts this motif. Altered binding can disrupt a motif that is represented in publicly available databases such as JASPAR [16], or a previously unknown TF binding motif predicted de novo directly from the ChIP-seq data [17]. By predicting motifs de novo, account is taken of TF-TF complexes that are pulled down in ChIP-seq experiments but are not reported in publicly available motif databases [18].

To predict ASBs accurately, methods should call variants that meet four criteria: (i) they match known genotypes [9, 11, 13], (ii) they meet stringent quality control and allele-dependent alignment of ChIP-seq reads [19], (iii) they lie within a ChIP-seq peak, and (iv) they disrupt (or strengthen) a TF’s binding motif when it reduces (or increases) its DNA-binding affinity. Previous ASB prediction methods meet some but not all four criteria [9, 10, 20, 21]. Furthermore, methods have not previously included a de novo motif discovery step, which limits the set of biologically-informative motifs available for investigation [19]. Prioritised ASBs that affect TF binding motifs can then facilitate genome-scale complex trait studies whose multiple testing burden is reduced due to the smaller number of prioritised variants.

Approaches such as AlleleDb [22], ADASTRA [10] and GVATdb [23] have been developed to catalogue ASBs across diverse TFs and cell types. Nevertheless, ASB catalogues are far from being complete, and reproducibility is a major challenge because these tools are neither executed end-to-end, nor easily applied across different computational platforms, nor containerised [24]. Our understanding of altered TF regulation across the genome would benefit from advances in pipeline development, and a large community effort [25] to build reproducible, portable, and containerised pipelines using workflow management systems such as nextflow [26] and snakemake [27].

To this end, we developed *baal-nf* (Bayesian analysis of allelic imbalance (baal) with nextflow (nf)), a new computational framework that infers ASBs by processing and quality controlling ChIP-seq data from the large number of studies that use genotyped cell lines, while accounting for known biases. Mapping these ASBs to known and de novo motifs then permits *baal-nf* to infer the TF mechanism by which binding is disrupted at these loci. To achieve our goals, we leverage nextflow to build an end-to-end pipeline, integrating tools for read and alignment quality control, alignment of high-quality reads, de novo motif discovery using NoPeak [17] and inference of ASBs using BaalChIP [9]. BaalChIP is a fully-Bayesian approach that infers ASBs from ChIP-seq data and, as shown by extensive simulation experiments, accurately corrects for biases due to copy number aberrations present in cancer cell lines.

*baal-nf* accounts for the aforementioned biological and technical biases. For (i) CNV and (ii) reference mapping biases, we implement the method described by de Santiago et al. [9] to infer ASBs, which explicitly models and accounts for these quantities during ASB inference. To reduce potential false ASB calls: (iii) we filter out PCR duplicate reads, (iv) we require ChIP-seq read coverage of heterozygous sites on both alleles and impose a strict genotyping quality threshold on these sites, (v) we remove ambiguously mapped reads, and (vi) we apply the method to cell lines, rather than more heterogeneous tissue samples.

We showcase *baal-nf* by its prediction of 298,783 ASBs from 558 TFs, and map these to 374 known and de novo motifs using publicly available ENCODE and ChIP-ATLAS data from 46 genotyped human cell lines. Among these are 1,935 high-quality, mechanistically well-understood ASBs. These are sites with strong evidence of binding, one of whose alleles both lowers a TF’s DNA-binding affinity and disrupts its binding motif. Consistent with their functionality, high-quality ASBs are more evolutionarily conserved across species, and are enriched for known trait associations and molecular quantitative trait loci (molQTL), supporting their prioritization in studies seeking causal trait-altering DNA variants.

## Results

### *baal-nf* workflow

Our aim was to create a workflow that infers ASBs, while correcting for sources of biological and technical bias, whose confidence is enhanced from their disruption of known or de novo inferred TF motifs. To apply this tool across a large set of studies, the workflow needed to be scalable, parallelizable and reproducible, with adaptability to new computational settings. To achieve this, we used nextflow, a workflow management system that allows implementation of complex data analysis workflows and that can be run easily across various computational platforms. Docker containers were implemented to obtain reproducible software environments at each step in the workflow. The *baal-nf* workflow implements BaalChIP, a Bayesian statistical approach that calls ASBs in cancer genomes [9], as detailed below. A summary of how *baal-nf* extends beyond BaalChIP and other methods is provided in Figure S1 (Additional file 1).

**Fig. 1:**
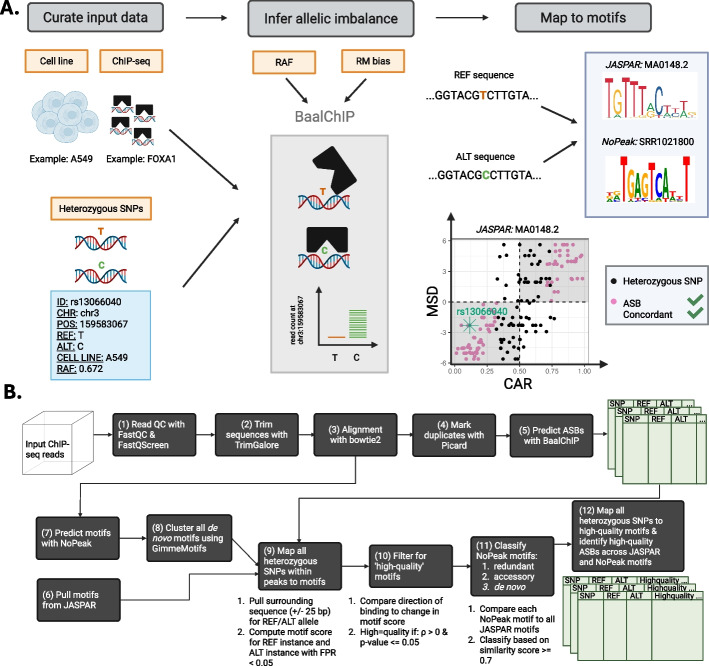
*baal-nf* workflow for identifying high-quality ASBs. **A** ChIP-seq data from genotyped cell lines is used to infer ASBs at heterozygous SNPs using BaalChIP, a beta-binomial model that takes into account biological bias due to copy number aberrations (through the reference allele frequency, RAF) and technical aspects such as reference-mapping (RM) bias. Identified ASBs are further characterised by mapping them to JASPAR and NoPeak motifs for a given TF. High-quality ASBs show concordance in the corrected allelic ratio (CAR) and motif score difference (MSD). These are the pink points in the bottom-right scatter plot defined by when a heterozygous SNP is (1) an ASB, and either (2a) CAR > 0.5 and MSD > 0 (i.e., the REF allele strengthens binding), or (2b) CAR < 0.5 and MSD < 0 (i.e., the ALT allele strengthens binding). **B** Bioinformatic workflow for processing and quality-controlling data, acquiring and identifying motifs, and determining how heterozygous SNPs are mapped to these motifs and further classified

*baal-nf* requires three sets of input data: (i) ChIP-seq FASTQ data from genotyped cell lines, (ii) reference allele frequencies (RAFs) for all heterozygous SNPs in each cell line-of-interest, and (iii) BED (Browser Extensible Data) files containing the ChIP-seq peak calls for a given sequencing run (Fig. 1A). FASTQ files are first quality controlled by filtering out reads with low sequencing quality or reads harbouring repetitive DNA, such as rDNA [28, 29] (Fig. 1B, steps 1–2). Remaining reads are then aligned to the human reference genome and duplicate reads marked as potential PCR duplications, before inferring ASBs with BaalChIP [9] (Fig. 1B, steps 3–5). BaalChIP then estimates the corrected allelic ratio (CAR), the preference of TF binding to either the reference or alternate allele at heterozygous sites after correcting for biological and technical biases (Fig. 1A) (Methods). A CAR estimate of 0.5 indicates no preference for TF binding to either allele; an estimate between 0.5 and 1 indicates higher TF affinity for the reference (REF) allele; and an estimate between 0 and 0.5 indicates higher affinity for the alternate (ALT) allele.

Hereon, *baal-nf* goes beyond BaalChIP functionality by mapping inferred ASBs to two types of TF motifs: known motifs for the experimentally-relevant TF (e.g., MA0148.2 for FOXA1), and de novo motifs that are enriched in the ChIP-seq read data (e.g., SRR1021800) (Fig. 1A). Known motifs for the relevant TF are downloaded from the JASPAR database [16] for use during the mapping procedure (Fig. 1B, step 6). De novo motifs are called from ChIP-seq reads using NoPeak, a k-mer-based motif discovery method that predicts motifs from global read distribution profiles, without requiring background ChIP-seq samples for motif discovery [17] (Fig. 1B, step 7) (Methods). NoPeak motifs are further clustered, combining information across highly similar motifs and removing redundancy within the NoPeak motif set.

Only heterozygous SNPs that lie within ChIP-seq peaks are considered at this stage, due to their strong evidence for TF binding, while we seek to derive a high-quality motif set. This set of heterozygous SNPs are mapped to all JASPAR and NoPeak motifs, resulting in motif scores for each of the REF and ALT alleles (Fig. 1B, step 9) (Methods). The difference in scores (the motif score difference, MSD) indicates whether the REF or ALT allele better matches the motif-of-interest.

Motifs that best explain allelic imbalance of TF binding are defined as “high-quality motifs”: those in which mapped alleles with higher binding affinity (inferred from ChIP-seq reads) show a significant tendency to be associated with stronger motif instance (i.e., with more positive MSD). More precisely, to be deemed “high-quality” a motif has a significant and positive correlation (Spearman’s correlation coefficient; SCC) between the CAR derived from BaalChIP and the calculated MSD, considering only heterozygous SNPs in peaks whose surrounding sequence match this motif well (Fig. 1B, step 10) (Methods).

To contextualise NoPeak motifs, we compare them against all known JASPAR motifs, defining a match between them based on motif similarity metrics. Motif similarity is assessed using the approach outlined in Grau et al. [30] and Kielbasa et al. [31] (Fig. 1B, step 11) (Methods). In this way, high-quality NoPeak motifs are classified into one of three groups: (i) redundant motifs – those that are good matches to known motifs in the JASPAR database for the ChIPped TF, (ii) accessory motifs – those that are good matches to known motifs in the JASPAR database that, however, are not linked to the ChIPped TF, and (iii) de novo motifs – those that are poor matches to JASPAR motifs. After identifying NoPeak motifs similar to, and thus redundant for, JASPAR motifs, these are discarded from further analysis.

Once the set of high-quality motifs is derived, *baal-nf* maps the entire set of heterozygous SNPs back onto these motifs (Fig. 1B, step 12). The workflow first identifies “high quality ASBs”: those whose altered TF binding within a ChIP-seq peak can be explained mechanistically by concordant disruption to a relevant, accessory or de novo TF binding motif. More specifically, these ASBs map to sequences matching a high-quality motif (for REF and ALT alleles) and that, additionally, show concordance between their direction of binding affinity (i.e., CAR) and their change in motif score (i.e., MSD) (Fig. 1A; highlighted as pink data points). Next, *baal-nf* identifies “low quality ASBs”: those that meet each of these criteria, except that they are located outside of a ChIP-seq peak. We did not wish to discard all ASBs outside of peaks because peak-calling algorithms vary in performance across different binding mechanisms [32]. All remaining ASBs not meeting these criteria or that did not map to a high-quality motif were defined as “unclassified ASBs”. Further below, we justify naming ASBs as “high quality” based on evolutionary information and associations to quantitative trait loci (QTLs).

### Redundant, accessory and de novo motifs found for FOXA1

To exemplify these steps, we describe next how we applied *baal-nf* to a single TF, namely Forkhead box A1 (FOXA1).

FOXA1 belongs to the FOXA subfamily of winged helix transcription factors that bind and open chromatin, thereby facilitating access for other transcription factors [33]. FOXA1 also binds cooperatively to other nuclear receptors [34, 35] to enable transcriptional activation and is associated with epigenetic regulation via DNA demethylation [36, 37]. FOXA1 binds DNA with high specificity, with binding affinity being highly dependent on minor variation of nucleotides within binding sites [38].

To predict FOXA1 ASBs we applied *baal-nf* to 156 ChIP-seq samples, 6 genotyped human cell lines and 270,587 heterozygous SNPs. Of 422,090 unique SNP-cell line pairs, 15,823 (3.7%) were predicted by BaalChIP to show allelic bias, with 420 of these replicated across two or more cell lines. Further, 9,214 (2.2%) lay within ChIP-seq peaks whose sequences were good matches to JASPAR and/or NoPeak motifs for FOXA1.

**Fig. 2:**
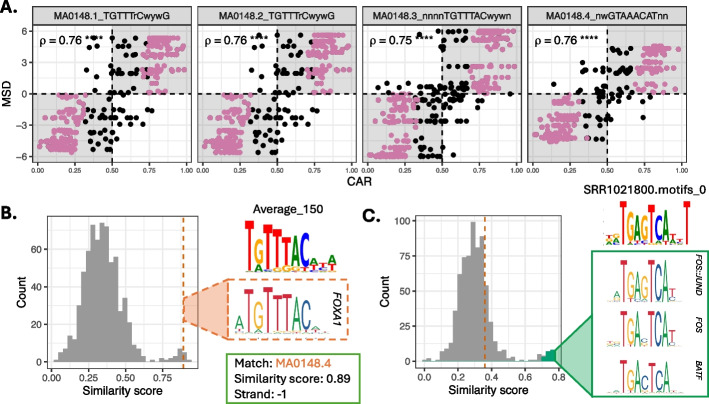
FOXA1 allele-specific binding inferred using *baal-nf*. **A** All four JASPAR motifs for FOXA1 are high-quality, showing a significant and positive Spearman’s correlation coefficient (ρ) between the CAR (x-axis) and MSD (y-axis). ρ is labelled on the plot, with significance levels **** representing a *p*-value < 2 × 10^–16^. Each scatter plot represents a unique JASPAR motif for FOXA1. **B** Redundant NoPeak motif identified for FOXA1 through *baal-nf*, where the NoPeak motif (“Average_150”) yielded a high similarity score of 0.89 (vertical dashed orange line) to MA0148.4, a FOXA1 JASPAR motif, when compared to similarity scores for all JASPAR motifs, shown here as a histogram (grey). **C** Accessory NoPeak motifs identified in FOXA1 ChIP-seq data, where the identified NoPeak motif (SRR1021800_motifs.0) yielded a high similarity score to FOS/JUN/BATF motifs (green cluster), and a low similarity score to FOXA1 JASPAR motifs (shown by dotted orange line)

All 4 JASPAR motifs for FOXA1 were high-quality motifs (i.e., showed concordant change in motif score and allelic ratio, (Fig. 2A)) and 11 of 135 NoPeak motifs were high-quality. High-quality JASPAR or NoPeak motifs tended to have higher information content than low-quality motifs (Additional file 1: Figure S2). Of the 11 high-quality NoPeak motifs, 1 was redundant, 2 were accessory motifs, and 8 were de novo. The redundant NoPeak motif (Average_150) showed a higher similarity score (0.89) to the FOXA1 JASPAR motif MA0148.4 than other JASPAR motifs (vertical dashed orange line, Fig. 2B).

One of the 2 accessory motifs (SRR1021800_motif_0) is a good match to FOS/JUN heterodimer and BATF motifs, but not to FOXA1 motifs (Fig. 2C, Additional file 2: Table S1). FOS, JUN and BATF are all members of the AP-1 family of transcriptional activators, known to form heterodimers with one another and to be in TF-coordinated complexes with FOXA proteins [39]. FOS has also been proposed to activate FOXA1 through the ERBB2 signalling pathway [40]. The other accessory motif (ENCFF774LQB_motif_0) is a good match to FOXP2, another member of the FOX family of TFs. These results demonstrate *baal-nf*’s ability to discover mechanistically-predictive TF binding motifs.

A total of 1,246 ASB events (7.9%) mapped to the 14 high-quality, non-redundant motifs (10 NoPeak, 4 JASPAR), with 224 being high-quality ASBs and 633 being low-quality ASBs (Methods). Across these 14 motifs, a median of 60 low-quality ASBs and 15 high-quality ASBs were identified (Additional file 3: Table S2). High-quality ASBs were discovered across JASPAR and NoPeak motifs in approximately equal measure, with 58% (129/224) mapped to JASPAR motifs, and 42% mapped to NoPeak motifs (16 to accessory motifs and 79 to de novo motifs) (Additional file 4: Table S3, Additional file 1: Figures S3-S4). This approximate doubling of high-quality ASB predictions for FOXA1 highlights the importance of including de novo motif discovery in this workflow. High-quality ASBs for FOXA1 were associated, on average, with 2.7 human physiological traits at a *p*-value threshold < 5 × 10^–8^, and 1.8 expression quantitative trait loci (eQTLs) (Additional file 1: Figure S5) (Methods).

### *baal-nf* predicts 1,935 high-quality binding variants across 558 TFs

**Fig. 3:**
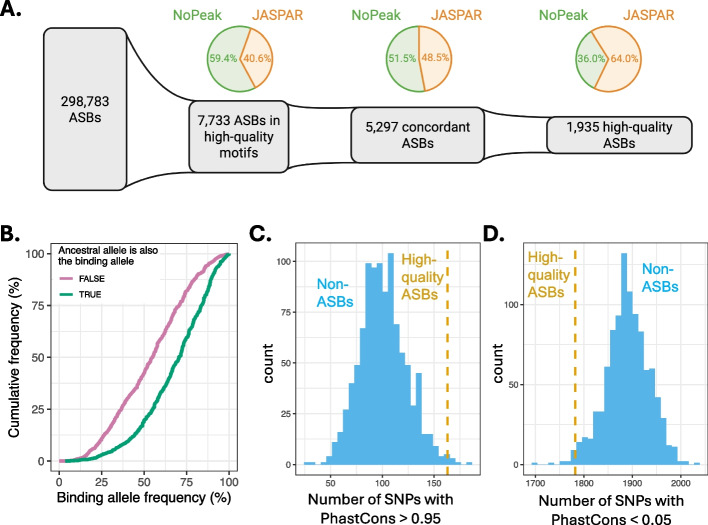
ASB set derived across 558 TFs and 46 genotyped cell lines, resulting in a subset of high-quality ASBs. **A** From the complete set of ASBs predicted by BaalChIP, 2.6% (*n* = 7,733) map within high-quality motifs, 1.8% (*n* = 5,297) display concordant behaviour in direction of inferred binding from BaalChIP (CAR) and disruption to a relevant motif (MSD), and 0.65% (*n* = 1,935) are deemed to be high-quality. **B** Empirical cumulative density function (ECDF) of frequencies of the binding allele in the non-Finnish European population from gnoMAD, split by whether the binding allele is the ancestral or derived allele. A Wilcoxon rank sum test comparing the means between the groups (FALSE and TRUE) show a significantly higher binding allele frequency when the ancestral allele is also the binding allele (TRUE), with a *p*-value of 4.62 × 10^–66^. **C** High-quality ASB sites (yellow dashed line) when compared to non-ASB sets (blue histogram) are significantly better conserved, as indicated by a higher number of SNPs with PhastCons scores > 0.95. **D** We find the opposite effect for non-conserved bases, with a significantly lower number of non-conserved bases present in the high-quality ASB set, represented by a PhastCons scores < 0.05

We applied *baal-nf* to 6,017 ChIP-seq data sets for 558 TFs and 46 genotyped cell lines (Additional file 5: Table S4), making up 1,056 TF-cell line groups. This resulted in 298,783 inferred ASBs across 531 of the 558 TFs, with 8,968 (3.00%) mapping to high-quality TF motifs. Of these, the binding change affinity for 5,063 (1.69%) was concordant with disruption to a relevant motif, and 1,935 (0.65%) passed the criteria (defined above) necessary to be assigned as high-quality (Fig. 3A, Additional file 6: Table S5, Additional file 7: Table S6). Among 3,014 JASPAR and NoPeak motifs investigated, 370 were high-quality (Additional file 1: Figure S6). Compared to low-quality motifs, high-quality motifs yielded consistently larger, positive Spearman’s correlation coefficients between CAR and MSD, indicating that they are more explicable of binding variation for investigated SNPs (Additional file 1: Figure S7).

High-quality ASBs (*n* = 1,935) were predicted by *baal-nf* for 86 out of 558 TFs in 46 genotyped cell lines. JASPAR motifs contributed most to this high-quality subset (average of 22 high-quality ASBs per TF), followed by NoPeak de novo motifs (average of 18 high-quality ASBs per TF) and NoPeak accessory motifs (average of 13 high- quality ASBs per TF) (Additional file 1: Figure S8). As with the FOXA1 example (above), inclusion of NoPeak motifs substantially expanded the set of high-quality ASBs, due to both accessory and de novo motifs being discovered across TFs (Additional file 1: Figure S9).

Variation in TF-binding affinity may not alter downstream molecular, cellular or organismal traits, and thus may not be functional [41]. To assess functionality of the high-quality ASB subset, we considered whether they had been subject to evolutionary selection, have known associations with human traits or are QTLs for transcript abundance or splicing (i.e., eQTL and sQTL, respectively).

For the 1,935 high-quality ASBs, we hypothesised that the allele associated with stronger TF binding affinity, i.e., the “binding allele”, would more often be the ancestral allele that has been retained in the population at higher frequency than the lower affinity, derived allele. Indeed, we discovered that when the binding allele is the derived allele then its population frequency is typically lower than when the binding allele is the ancestral allele (*p*-value = 4.6 × 10^–66^) (Fig. 3B). This is consistent with positive selection of the binding allele and/or negative selection of the lower affinity allele. Previous studies have shown a similar effect when investigating ASBs across 94 TFs and 2 patient donors [42].

Next, we showed that high-quality ASB sites tend to be evolutionarily well conserved. For this analysis, we generated 1000 comparator sets of “non-ASBs”, defined as randomly sampled high read coverage heterozygous SNPs that, when tested by *baal-nf*, failed to show significant allelic bias in any cell line, and for any TF (Methods) (Additional file 8: Table S7). We define high read coverage here as ≥ 100 reads mapping to the region containing the SNP. SNPs in these non-ASB sets were matched on minor allele population frequencies to those of high-quality ASBs, and had the same number of SNPs as the high-quality set (Methods).

SNP positions in both groups were scored for evolutionary conservation across 30 eutherian mammals using PhastCons [43]. PhastCons scores range between zero – no conservation of that SNP across the species-of-interest – and one, indicating complete conservation. The number of highly conserved bases (PhastCons score > 0.95) was significantly larger in the high-quality ASB set (empirical *p*-value = 8.0 × 10^–3^), and the number of non-conserved bases (PhastCons score < 0.05) was significantly lower in the high-quality ASB set (empirical *p*-value = 1.4 × 10^–2^) (Fig. 3C-D).

Compared with non-ASBs, we found that high-quality ASBs also had: (i) a significantly higher number of known variant-human trait relationships (*p*-value = 1.6 × 10^–3^), (ii) more eQTLs (*p*-value = 1.2 × 10^–9^), and (iii) a higher maximum OpenTargets V2G (Variant-to-gene) score when associating each variant with a downstream gene (*p*-value = 1.0 × 10^–9^), but no significant difference in the number of sQTLs (*p*-value = 0.9) [44]. As expected, minor allele frequencies were not significantly different between our non-ASB set and high-quality set, mitigating ASB discovery biases (“count100” in Additional file 1: Figure S10A). Findings were also robust to different count thresholds used to define high coverage SNPs for the random samples of non-ASBs (Additional file 1: Figure S10B-E, Figure S11). Both evolutionary conservation analyses and trait/QTL-association analyses were consistent when using a more lenient minimum read coverage threshold of 50 to derive our 1000 non-ASB comparator sets (results for “count50” are shown in Additional file 1: Figures S9B-D and S10).

With the same analyses, “low-quality ASBs” were found not to be more evolutionarily conserved compared to their non-ASB sets (Additional file 1: Figure S12). They were, however, significantly associated (*p*-value = 2.2 × 10^–3^) with a higher number of human traits at a *p*-value threshold < 5 × 10^–8^, but not for the numbers of colocalised eQTLs or sQTLs, or the maximum V2G score (Additional file 1: Figure S13, Additional file 9: Table S8, Additional file 10: Table S9).

In summary, by applying *baal-nf* across a large set of TFs, we have generated a new database of 298,783 ASBs and have identified a subset of 1,935 high-quality ASBs. For these high-quality ASBs, the differential binding mechanism, inferred using high-quality motifs, provides high confidence for functionally significant altered binding at these loci. When compared to non-ASB SNPs, this high-quality ASB set exhibited greater conservation across species, a higher number of known associations with traits, and greater colocalization with molecular QTLs.

### Catalogues of ASBs are far from complete

**Fig. 4:**
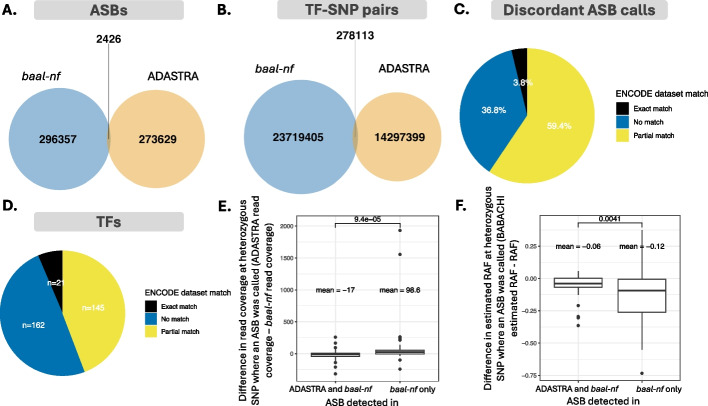
Feature-level differences in ASB calling across ADASTRA and *baal-nf.* 296,357 ASBs (99%) predicted by *baal-nf* were not previously reported by the ADASTRA resource, due largely to minimal overlap in the sets of SNP-TF pairs investigated in both approaches. Additional differences will be due to ADASTRA not requiring heterozygous SNPs to be genotyped prior to its calling of ASBs, as well as differing input ChIP-seq data. We find that **A** only 2,426 ASBs are identified in both databases, meaning that 296,357 predicted by *baal-nf* (blue) have not been previously reported by ADASTRA (orange). **B** TF-SNP pairs investigated by both methods are mostly distinct, accounting for much of the difference in their discovered ASBs. Of TF-SNP pairs that were investigated by both, only **C** 3.8% of discordant calls between the two methods used the same input data, which is likely to account for further differences. ASBs are shown from these methods when they both used the same (black) or a partial set (yellow) of ENCODE project IDs. No match (blue) indicates ASBs for which input data sets for *baal-nf* and ADASTRA were completely different. **D** As panel C, but for numbers of TFs assessed by the two methods using the same ENCODE datasets. **E** For 21 TFs assessed by both methods using the same data, 14,194 SNP-TF pairs were investigated. Across these 14,194 TF-SNP pairs TFs and the 102 ASBs called by ADASTRA, ASBs that were called only by ADASTRA and not *baal-nf* (“TRUE”) tended to have significantly higher read coverage at that SNP, compared to ASBs that were detected by both. **F** Across these 21 TFs and the 533 ASBs called by *baal-nf*, the reference allele frequency (RAF) was significantly different to the background allelic dosage estimated by in ADASTRA

Finally, we compared predicted ASBs from *baal-nf* to known ASB databases, ADASTRA [10], GTRD [45], GVATdb [23], and AlleleDb [22]. ASB incompleteness is evident from the small overlap between *baal-nf*- and ADASTRA-derived ASBs (Fig. 4A, Methods). Incompleteness is due to substantial differences in the TF-SNP pairs (Fig. 4B), and the input ChIP-seq datasets (Fig. 4C-D) being tested. With the availability of the *baal-nf* end-to-end workflow, this incompleteness can now be reduced.

Across ADASTRA, GTRD, GVATdb and AlleleDb, most differences were due to mismatching TF-SNP pairs being tested across the databases (Supplemental Material, Methods). When TF-SNP pairs matched, 95.3–100% of ASB calls were concordant with *baal-nf* (Additional file 1: Figures S14-S17, Additional file 1: supplemental note)*.* Discordant results could result from different ChIP-seq datasets in different cell lines (Fig. 4C-D), different read coverage at heterozygous SNPs (Fig. 4E), and differences in estimating CNVs (Fig. 4F). A more detailed comparison to these databases is presented in the supplemental note (Additional file 1).

In summary, few TF-SNP pairs have been investigated, or called as ASBs, by multiple methods. Human genomics research remains far from revealing the full complement of TF binding QTLs which will require application of scalable, reproducible ASB calling computational pipelines such as *baal-nf*.

## Discussion

Identification of trait-causal variants would yield insights into fundamental biology that could aid development of therapeutic interventions. Nevertheless, such variants are challenging to identify due to linkage disequilibrium, whereby neighbouring SNPs are often co-inherited, leading to significant variant-trait associations that mainly reflect correlation, not causation. Assigning molecular mechanisms to SNPs is critical for prioritization of variants that are truly causal [46–49]. Extensive cataloguing of molecular QTLs is required to refine the evidence for how finely-mapped SNPs can mechanistically explain trait variation.

Substantial efforts have been made to identify and annotate cis- and trans-expression QTLs and splicing QTLs through databases like eQTLGen [50], GTEx [51], eQTL Catalogue [52], single-cell eQTLGen Consortium [53], as well as integrated platforms such as QTLbase2 [54] and OpenTargets [44, 48]. Less effort has been expended on TF binding QTLs: such resources are scarce and are seldom integrated into these platforms. Databases for ASBs such as AlleleDb [22], GTRD [45] and ADASTRA [10], with extensions such as ANANASTRA [55], have sought to address this gap. However, these tools are not easily applied to new datasets and do not include the processing of raw ChIP-seq datasets or de novo motif discovery. Here, we sought to help narrow the information gap and provide a tool for future large-scale identification of new and high-quality ASB datasets.

*baal-nf* allows for rapid and robust inference of ASBs and a high-quality subset that can be prioritised in genomic studies. In this study, we have applied *baal-nf* to genotyped cell lines. To retain performance, application of *baal-nf* to tissue-level data would require cell type deconvolution and extension of existing benchmarking on cell lines to tissues [9]. Here, we have only applied *baal-nf* to ChIP-seq data, but it is equally extendable to other high-throughput sequencing data sets, for example from CUT&RUN or CUT&TAG, or even RNA–protein based approaches such as cross-linking and immunoprecipitation followed by sequencing (CLIP-seq) [56]. Motif discovery is expected to lack power if *baal-nf* is applied to open chromatin data, such as from ATAC-seq or DNAse-seq, due to the multiplicity of TFs involved. We have also applied *baal-nf* here only to single nucleotide variants, but it is equally applicable to short insertion/deletion variants.

*baal-nf* is an open-source software, available on GitHub, which can be used to derive new ASB sets and high-quality ASBs from ChIP-seq datasets. It supports end-to-end processing of raw sequencing data, inference of ASBs and characterization of these ASBs with respect to binding mechanism. For large-scale application, a high-performance compute (HPC) cluster is recommended for efficient parallelization of the workflow. A major challenge in genomic research is the ability to make research portable and reproducible [24]. To achieve this, we rely on nextflow and singularity, allowing large-scale implementation of this tool with stable and reproducible software environments [26]. Limitations of *baal-nf* include BaalChIP’s reliance on the hg19 genome assembly, and its current dependence on ChIP-seq data. Furthermore, non-JASPAR reference motifs are not currently integrated into the workflow. This tool can be extended in the future for compatibility with additional genome assemblies, motif databases, and other sequence-based methods that measure allelic imbalance.

We hope that others will add to this initial discovery set of high-quality ASBs by applying *baal-nf* to further data sets. All such high-quality ASBs will be useful when prioritizing non-coding regulatory variants as causal of complex trait variation and disease risk.

## Conclusions

Of the 298,783 ASBs reported in this study, 0.65% (*n* = 1,935) of these were “high-quality”. We proposed this high-quality set as strong candidates for causal trait-altering DNA variants, as demonstrated by enrichment for known variant-trait relationships, colocalization with gene expression QTLs and strong evolutionary conservation. Although limited in size, this high-quality set’s variants are mechanistically well-understood and have evidence for altered binding across multiple datasets. This is because the method requires: (i) strong evidence for the called genotype at heterozygous SNPs, (ii) strong evidence for allelic imbalance, (iii) orthogonal evidence for altered binding by disruption of a TF binding motif, and (iv) strong evidence for binding by residing within a ChIP-seq peak. By proposing a high-quality set, we enable researchers to prioritise these variants in large-scale genomic studies.

## Methods

### Input data to *baal-nf*

ChIP-seq data used in this study was acquired from ENCODE and ChIP-ATLAS. This contained: (1) raw FASTQ files for ChIP-seq, (2) BED files containing called peaks for the same experiments, and (3) B allele frequencies (BAFs) for all cell lines investigated. BAFs acquired from ENCODE are initially quality controlled to filter on genotyping quality (> 0.95 call rate), which handles potential false variant calls that would impair ASB inference. ENCODE or ChIP-ATLAS accession IDs are listed in Table S4 (Additional file 5).

### Read QC, alignment and inferring ASBs

For *baal-nf*, read quality is assessed using FastQC (version v0.11.9) and FastQScreen (version v0.14.0) and then trimmed using TrimGalore! (version v0.6.7), filtering out any reads lying below a minimum quality threshold (default parameters in TrimGalore!)^19,20^. High-quality reads are aligned using bowtie2 [57] (version v2.3.5.1) to hg19, the human reference genome used in BaalChIP, duplicate reads are marked with Picard (version v2.23) [58], and aligned BAM files are used to infer ASBs using BaalChIP [9] (custom version modified from v1.1.1; provided at https://github.com/BAAL-NF/BaalChIP). BaalChIP models allelic counts with a beta-binomial model, correcting for CNVs using the RAF as a prior, and correcting for reference Mapping (RM) bias, which is estimated directly from the data. An ASB is called only if the highest posterior density interval for the fraction of reads mapping to the REF allele does not include a value of 0.5. Heterozygous SNPs predicted to be ASBs will have a value of “True” set in the output column “isASB”. The corrected allelic ratio (CAR) will also be reported for each SNP, which describes preference in binding to either the REF (CAR > 0.5) or ALT (CAR < 0.5) allele, after correcting for the RAF and RM bias.

### Significance testing for ASBs

ASBs are inferred using BaalChIP [9], which is a fully-Bayesian approach to infer ASBs. Briefly, this model uses the allelic ratio (ratio of the number of REF reads to number of ALT reads) at a given SNP as the likelihood of binding to the REF allele, explicitly modelling the RM bias and using the RAF as a prior. On the resulting posterior distribution of allelic imbalance, a Metropolis–Hastings algorithm is employed to approach this distribution. An ASB is called if the highest posterior density interval (defined at the 95% level) does not contain the value 0.5. This approach was shown, through extensive simulations, to be robust to false positive ASB calls, particularly those attributed to CNVs that are present in cancer cell lines, and in their approach, are explicitly modelled using the RAF. In our implementation, we allow the credible interval threshold to be user-defined, with a default of 0.95. All ASBs that pass this significance threshold are assigned a value of “True” to the column “isASB”.

### Predicting motifs with NoPeak

Aligned BAM files are converted to BED files using bedtools (version v2.29.2), and NoPeak [59] is used to compute score profiles for each k-mer of length 8, the NoPeak default setting. Briefly, a score profile is computed by estimating the density of reads which include a given k-mer and computing the distance from the beginning of that read to the k-mer of interest. Profiles that are consistent with TF binding have an increased frequency of reads over the k-mer sequence. All k-mer profiles that indicate TF binding are combined based on sequence similarity, resulting in predicted motifs per-ChIP-sequencing sample. Low k-mer motifs derived from fewer than 10 k-mers are excluded from subsequent steps as they are generally low-complexity and not indicative of true binding motifs.

## Identifying non-redundant NoPeak motifs

Predicted NoPeak motifs are defined per-ChIP-seq sample, leading to likely redundant motifs across multiple samples ChIPped for the same TF. To remove redundant motifs, all NoPeak motifs discovered for a given TF are clustered using GimmeMotifs (version v0.17.2) [60], using de Bruijn sequences to quantify motif similarity (cluster_motifs() function: trim_edges = True, metric = “seqcor”, threshold = 0.7, combine = “mean”). A minimum threshold of 0.7 is chosen based on the analysis using the “seqcor” metric in Grau et al. [30]. This scores each motif against one another, trimming low information content bases at the edge of the motif, and clustering motifs together that have a similarity score greater than 0.7. Each motif cluster is then averaged across the length of the motif to obtain a final, clustered motif. The resulting motifs from NoPeak are named after the ChIP-seq sample in which it was discovered or, if clustered, are named Average_n, where n is a number randomly generated by GimmeMotifs.

### Mapping heterozygous SNPs to JASPAR and NoPeak motifs

Heterozygous SNPs are mapped to JASPAR and NoPeak motifs using GimmeMotifs (version v0.17.2) [60]. Sequence instances for the REF and ALT allele for each heterozygous SNP are derived by extracting ± 25 bp around the position of the relevant SNP from the human reference genome assembly (here, hg19). Each instance is scored against a motif-of-interest (the motif score), which is represented as the log-odds of belonging to that motif compared to a random background sequence from the same reference genome, with a false positive rate less than 5 × 10^–2^. The motif score difference (MSD) is computed by taking the difference between the REF motif score and the ALT motif score. An MSD of zero indicates that the REF allele does not disrupt the motif-of-interest compared to the ALT allele; a positive MSD indicates that the REF allele is more likely to belong to the motif-of-interest; and a negative MSD indicates that the ALT allele is more likely to belong to the motif-of-interest. “High-quality” motifs are determined by computing the Spearman’s Correlation Coefficient (SCC; $\rho$) between the CAR and MSD across all heterozygous SNPs that map to a given motif, and then filtering for motifs with a significant, positive SCC (*p*-value < 0.05). Only such motifs are considered when identifying high-quality ASBs.

### Characterizing NoPeak motifs

To assess similarity of NoPeak motifs to known JASPAR motifs, we compute a similarity score for each NoPeak motif compared to all known JASPAR motifs (organism: Homo sapiens) using GimmeMotifs (version v0.17.2). We follow the approach outlined in Grau et al. [30] and Kielbasa et al. [31] using de Bruijn sequences to compute a similarity score. Each step along the chosen de Bruijn sequence, representing every k-mer of length k (k = 7, the default provided in GimmeMotifs), is scored against the first motif and second motif, resulting in two vectors of motif scores, termed score profiles. If the motifs are highly similar, then these score profiles are highly correlated, and have a strong, positive Pearson correlation coefficient (PCC). The PCC between score profiles is computed for every possible offset of the two motifs as well as the reverse complement, and the maximum score (termed “similarity score”) is taken. NoPeak motifs with a similarity score greater than 0.7 to a known JASPAR motif will either be classified as (1) redundant: the NoPeak motif matches the canonical JASPAR motif for the TF-of-interest, in which case it is removed from further analysis, or (2) accessory: the NoPeak motif matches a known JASPAR motif that is not the canonical binding motif Kielbasa et al. [31].

### ASB subsets – defining high and low-quality ASBs

Concordant ASBs are derived by filtering for all ASBs that map to a high-quality motif, and either (1) the CAR is > 0.5 and the MSD > 0 or (2) the CAR < 0.5 and the MSD < 0. This subset is further characterised by whether or not the SNP lies within a ChIP-seq peak. ASBs that lie within ChIP-seq peaks are termed “high-quality” and those that lie outside of ChIP-seq peaks are called “low-quality”. As the same SNP can map to multiple motifs, we define an order in which to call SNPs concordant by first prioritizing those that lie within (i) JASPAR motifs, then (ii) NoPeak accessory motifs and then (iii) NoPeak de novo motifs. This logic is applied to arrive at the results compiled in Table S5 (Additional file 6) and should be used to derive high-quality ASB sets. Individual motif-mapping results (for example, in Additional file 4: Table S3) should be used to inspect motif-level concordance for each motif individually.

### Summarizing ASB sets

Throughout this manuscript’s main text, ASB summary numbers for 531 TFs represent unique TF-SNP calls. Consequently, ASBs that are replicated across multiple cell lines for the same TF-SNP pair are counted once. For example, Table S5 (Additional file 6) contains all 301,587 ASBs predicted by *baal-nf*, yet by applying the following operations using the R package dplyr (v1.1.4): tables5% > % distinct(ID,tf) % > % nrow(), we arrive at the non-redundant set of *n* = 298,783 ASBs. For high-quality ASBs, the equivalent operation is: tables5% > % filter(ASB_quality = = "High") % > % distinct(ID,tf) % > % nrow(), yielding the high-quality set of 1,935 ASBs in Table S6 (Additional file 7). The 224 FOXA1 high quality ASB events reported in the main text is slightly different, as it includes the same SNP if it was discovered in multiple cell lines, and can be derived by application of tables5% > % filter(tf = = "FOXA1",ASB_quality = = "High",!is.na(ASB_quality)) % > % nrow().

### Deriving non-ASB sets

To compare sets of “non-ASBs” to the high-quality ASB set, all assessed heterozygous SNPs across all 46 genotyped cell lines were inspected, and any SNP (specifically rsID) for which an ASB was called by BaalChIP was removed from this set. This resulted in a final set of analysed SNPs for which an ASB was not called in any cell line assessed across all 558 TFs. Each heterozygous SNP in this set was then required to be “high coverage” (number of reads ≥ 100 at its site) to ensure sufficient coverage to confidently call this site as non-ASB. We also evaluated this workflow at minimum read threshold of 50 (“count50” in Additional file 1: Figures S9-S11). Every SNP in this non-ASB sample set was queried using the ENSEMBL REST API to pull the gnoMAD minor allele frequency for the non-Finnish European population. Non-ASBs’ rsIDs were randomly sampled with replacement from sets matched on minor allele frequency (MAF) within a 5% relative threshold, and this process was repeated across all high-quality SNPs to form a sample size of 2,405 non-ASB SNPs, a number matching the count of ASBs found in the high-quality set, including SNPs that were replicated across cell lines for the same TF. This sampling procedure was repeated 1000 times to generate 1000 non-ASB sets. A single “median” non-ASB set was also derived by selecting the SNP with the median MAF across the 1000 sampled datasets for each SNP in the total set of MAF-matched SNPs (*n* = 2,405). This results in a single “median” non-ASB dataset matching the SNP set size of 2,405. This median set was used for querying statistics from OpenTargets, including the number of trait associations found at a *p*-value threshold of 5 × 10^–8^, the number of colocalised eQTLs and sQTLs, and the maximum V2G score for that variant. This same process was repeated for low-quality ASBs, with a set size of 3,627 to derive new non-ASB reference sets matched on MAF. See Figure S18 (Additional file 1) for a flow chart of this workflow.

### Evolutionary and functional genomics analysis of SNP groups

PhastCons scores for all SNPs assessed in 30-way bigwig files (for GRCh38) were pulled from UCSC [61] and scores computed for SNPs in high-quality ASB and non-ASB groups using ENSEMBL’s variant effect predictor VEP (version 112) [62]. Variant-trait and variant-gene relationships were determined for each SNP group by querying OpenTargets Genetics [44, 48] using their GraphQL API. For each variant, every variant-trait and variant-gene relationship record was pulled, and the following values were computed for each variant: (1) number of traits that passed a significance threshold of *p* < 5 × 10^–8^, (2) number of colocalised eQTLs, (3) number of colocalised sQTLs, and (4) maximum V2G score. Additional information was queried using the ENSEMBL REST API, including information about which allele was ancestral, as well as minor/major alleles in a population-of-interest and their respective frequencies in that population. Here, we used the gnoMAD non-Finnish European population, and in cases where this data was not available, the 1000Genomes GBR population. See Figure S19 (Additional file 1) for a flow chart of this workflow.

### Comparison with ADASTRA

Results from the latest release of ADASTRA (Mabel; v6.1) were acquired from https://adastra.autosome.org/mabel/downloads (specifically, those with one file per TF). All ADASTRA results were compiled into a single CSV file and filtered for those where the REF or ALT allele was significant after FDR correction [(fdrp_bh_ref < 0.05) | (fdrp_bh_alt < 0.05)]. ASBs and SNPs were compared across databases at the SNP-TF pair-level by creating a unique ID for each TF-SNP pair and investigating overlapping IDs. For the 278,113 SNP-TF pairs assessed in both methods, concordant calls were those that were either (i) called as an ASB in both approaches (*n* = 2,426) or (ii) called as a non-ASB in both (*n* = 262,489). Discordant calls were SNPs that were called as an ASB in *baal-nf* and a non-ASB in ADASTRA (*n* = 8,191), or an ASB in ADASTRA and a non-ASB in *baal-nf* (*n* = 5,007). Concordant ASBs in matching TF-SNP pairs in the two databases were derived by filtering for motif_conc = = “Concordant” in ADASTRA and ASB_quality = = “High” in *baal-nf*. To assess matching input data between ADASTRA and *baal-nf*, input samples were downloaded from ADASTRA at https://adastra.autosome.org/assets/exps/ADASTRA_GTRD_exps.bill_cipher.tsv. Next, ENCODE project IDs were compared from this table to Table S4 (Additional file 5) presented in this manuscript. If: (i) the same ENCODE datasets were used for a given TF, this was defined as an “exact match”, (ii) some ENCODE datasets overlapped, this was a “partial match”, or (iii) no ENCODE datasets were the same across the two databases, this was defined as “no match”. For exactly matching TFs (*n* = 21), comparative features were generated on matching TF-SNP pairs, comparing differences in read coverage (“total_cov” (ADASTRA) minus “Total.counts” (*baal-nf*)) and estimated RAF (“BABACHI_estimated_RAF” (ADASTRA) minus “RAF”). BABACHI is the method used by ADASTRA to estimate background allelic dosage (BAD), from which the RAF can be estimated: RAF = 1/(mean_BAD + 1).

### Comparison with GVATdb

GVATdb results were downloaded from https://renlab.sdsc.edu/GVATdb/search.html. To compare to *baal-nf*, results were matched on SNP (rsid), REF, ALT and TF. ASBs in GVATdb are defined as pbSNPs, as reported in their study, at a *p*-value threshold < 0.01. Both ASB and non-ASB calls were compared across the two approaches by intersecting SNP/REF/ALT/TF IDs that matched between them. Candidate ASBs tested in both were found by checking the intersection of SNP/REF/ALT/TF IDs that were tested in both (*n* = 3,170).

### Comparison with AlleleDb

Motif-concordant ASB calls in Supplementary file 7 were downloaded from https://archive.gersteinlab.org/proj/alleledb/downloads. Both AlleleDb and *baal-nf* were filtered to include only TF-cell line pairs that matched between the two databases. This included CTCF/GM12891, CTCF/GM12892, CTCF/GM12878, YY1/GM12892, BATF/GM12878, and PAX5/GM12878. These were merged with *baal-nf* ASB calls by matching by cell line, TF and SNP (denoted by CHR:POS:REF:ALT). Concordant calls were determined by intersecting SNP/TF/cell lines that were found in both databases. Non-ASBs were not reported in AlleleDb, so only ASBs from this database could be assessed for concordance with *baal-nf* results.

## Supplementary Information

Additional file 1: Supplementary Figures. All supplementary figures (ie. Figure SX) referenced in this manuscript, as well as the supplemental note.

Additional file 2: Table S1. Accessory motif matches to JASPAR database for FOXA1 NoPeak motifs.

Additional file 3: Table S2. Summary statistics for different SNP groups mapping to all 15 high-quality motifs found for FOXA1.

Additional file 4: Table S3. *baal-nf* output for all heterozygous SNPs tested in FOXA1 run across 156 ChIP-seq samples.

Additional file 5: Table S4. Sample list & accession numbers from ENCODE/ChIP-ATLAS for 558 TF run.

Additional file 6: Table S5. *baal-nf* output for all ASBs called across 558 TFs and 6017 ChIP-Seq samples.

Additional file 7: Table S6. High-quality ASB set discovered by *baal-nf* with ENSEMBL/OpenTargets annotation.

Additional file 8: Table S7. Median non-ASB comparator set for high-quality ASBs with ENSEMBL/OpenTargets annotation.

Additional file 9: Table S8. Low-quality ASB set discovered by *baal-nf* with ENSEMBL/OpenTargets annotation.

Additional file 10: Table S9. Median non-ASB comparator set for low-quality ASBs with ENSEMBL/OpenTargets annotation.

Additional file 11: Table S10. Motif metadata across all investigated motifs and 558 TFs.

Additional file 12: Table S11. Accessory NoPeak motifs discovered by *baal-nf*.

## Data Availability

All data generated or analysed during this study are included in this published article or are from publicly available sources. All supplementary tables can be downloaded from the Open Science Framework (OSF) at https://osf.io/rwjec/ under the project osf.io/rwjec (v0.1.0; DOI 10.17605/OSF.IO/RWJEC) [63]. Input data for FASTQ and BED files were downloaded from ENCODE (at https://www.encodeproject.org/) [64] & ChIP-ATLAS (at https://chip-atlas.org/) [65]. In total, there were 272 unique publications linked to the ChIP-seq databases analysed in this study, and the citations and associated accession IDs are included in Table S4 (Additional file 5). Additional ChIP-seq FASTQ and BED files were used for the vitamin D receptor (VDR) from Gallone et al. [66]. Results from this study are contained within Tables S5, S6, and S10. Table S5 (Additional file 6) contains all 298,783 predicted ASBs across 558 TFs, Table S6 (Additional file 7) contains all high-quality ASBs and queried information from ENSEMBL and OpenTargets. Table S10 (Additional file 11) contains all motifs explored in this study with associated metadata for each TF, and Table S11 (Additional file 12) contains all high-quality accessory motifs discovered by baal-nf. All SNP-cell line mappings, including characterization with respect to high-quality motifs, are deposited on OSF under project osf.io/rwjec in the folder “database”, separated by transcription factor. All code is deposited on GitHub at https://github.com/BAAL-NF/baal-nf and is publicly available [67]. baal-nf version implemented in this manuscript is 0.9.4. We have additionally created a vignette to outline how baal-nf might be run, including providing input data, as well as example outputs from this run (hosted at https://github.com/BAAL-NF/vignette) [68]. Many repositories contributed to the analysis presented here, including those with custom python packages for SNP-motif mapping [69, 70], a custom version of BaalChIP [71], and a nextflow pipeline for the evolutionary and functional genomics analysis comparing non-ASBs to high-quality ASBs [72]. All figures were generated using bioRender [73]. All GitHub repositories are available under an open access MIT license.
