# Supplementary material for: baal-nf identifies motif-disrupting variants that decrease transcription factor binding affinity
Source: Genome Biol. 2026 Jan 13;27:24. doi: 10.1186/s13059-025-03916-9 (PMC12888418; doi:10.1186/s13059-025-03916-9)
Supplement: Supplementary file 1 — Additional file 1: Supplementary Figures. All supplementary figures (ie. Figure SX) referenced in this manuscript, as well as the supplemental note. [file 13059_2025_3916_MOESM1_ESM.docx]

Additional File 1: Supplementary figures and supplemental note

**Figure S1: Comparison of *baal-nf* to existing ASB calling methods**. *baal-nf* offers ten advantages over existing methodologies. It is packaged into a nextflow implementation that is (i) portable and (ii) reproducible; it implements (iii) all pre-processing, QC and alignment steps required for subsequent ASB inference; and (iv) it integrates BaalChIP for ASB inference into this workflow, whose performance has been verified by extensive simulation experiments. *baal-nf* also: (v) performs *de novo* motif discovery on the ChIP-seq data itself, expanding the set of biologically-relevant motifs beyond those in publicly available databases, and then (vi) maps all investigated SNPs onto the joint set of publicly-available and predicted motifs. It can be applied to (vii) single ChIP-seq experiments, or to (viii) large-scale parallelised execution across thousands of datasets. Lastly, *baal-nf* accounts for known (ix) biological biases (such as CNVs) and (x) technical biases that can confound ASB inference. However, *baal-nf* requires genotyping and peak data which, if absent, limits its application.

**Figure S2: Properties of high-quality motifs for FOXA1.** Empirical cumulative density frequency (ECDF) plot showing information content of high- (green) versus low- (orange) quality motifs found for FOXA1. Information content (x-axis) is measured in bits.

**Figure S3: Redundant and accessory motifs discovered for FOXA1.** Scatter plots for **(A)** redundant and **(B)** accessory motifs found for FOXA1 via NoPeak. High-quality ASBs are coloured in pink. CAR (x-axis) is the corrected allelic ratio calculated by BaalChIP for a heterozygous SNP lying within a ChIP-seq peak; MSD (y-axis) is the motif score difference for each SNP mapped to the labelled motif.

**Figure S4: *de novo* motifs found for FOXA1 via NoPeak**. High-quality ASBs are coloured in pink. CAR (x-axis) is the corrected allelic ratio calculated by BaalChIP for a heterozygous SNP lying within a ChIP-seq peak; MSD (y-axis) is the motif score difference for each SNP mapped to the labelled motif.

**Figure S5: Number of associated traits & eQTLs for high-quality ASBs predicted for FOXA1.** These values were obtained by querying for the variant in OpenTargets Genetics and summarizing statistics for each category – **(A)** Number of trait associations that passed a p-value threshold of 5 x 10^-2^, **(B)** Number of trait associations that passed a p-value threshold of 5 x 10^-8^, and **(C)** Number of colocalised eQTLs.

**Figure S6: Number of high and low-quality motifs detected across 558 TFs.** Transcription factors are shown on the x-axis, and the number of high-quality (green) versus low-quality (orange) motifs for each of these TFs is shown on the y-axis.

**Figure S7: Spearman’s correlation coefficient for high- (green) versus low- (orange) quality motifs in JASPAR and NoPeak motifs investigated across all 558 TFs.** **** indicates a p-value < 2 x 10^-16^ with p-values computed using a Wilcoxon rank sum test.

**Figure S8: Breakdown by motif group for high-quality ASBs.** Expanding our motif set to include accessory and *de novo* motifs from NoPeak substantially increases the number of high-quality ASBs.

**Figure S9: Numbers of NoPeak motifs detected across the 558 TF run.** A range of NoPeak accessory and *de novo* motifs are discovered, with more *de novo* motifs than accessory motifs found overall.

**Figure S10: Trait/QTL associations for high-quality ASBs compared to non-ASB comparator sets.** **(A)** As these non-ASB sets were matched on minor allele frequency (MAF), we see no significant difference between the MAFs for the high-quality ASB and non-ASB sets with p-values of 0.9 and 0.9 for the count 50 and count 100 thresholds, respectively. Non-ASB sets thresholded above 50 counts are enriched for **(B)** known variant-trait associations, and **(C)** colocalised eQTLs, but not for **(D)** sQTLs, although they tend to have **(E)** higher maximum V2G scores. Data is queried from OpenTargets for the median non-ASB set for counts threshold of 100 and 50 and the high-quality set. The median non-ASB set was defined by choosing the SNP with the median MAF across all 1000 sampled sets, for each SNP (Methods). Note that the y-axis in plots **(B-E)** is log10-scaled.

**Figure S11: High-quality ASBs are more conserved than non-ASB sets across varying counts thresholds.** This figure details the same analysis using PhastCons scores shown in Figure 3 but for non-ASB sets with a minimum counts threshold of 50. **(A)** High-quality ASBs (dashed yellow line) harbour more conserved SNPs compared to count 50 non-ASB sets (green). **(B)** High-quality ASBs (dashed yellow line) have fewer non-conserved SNPs compared to count 50 non-ASB SNPs. PhastCons scores are pulled from the 30-way UCSC track for non-ASBs (green) and high-quality ASBs (yellow). The set of SNPs used to derive the sampled non-ASB sets was relaxed to a slightly lower read coverage minimum threshold of 50 counts. More specifically, the region containing the investigated SNP must not be called as an ASB in any cell line, for any TF, and have at least 50 reads mapping to it (Methods). This is different to the analysis in the main figure which used a minimum count threshold of 100 reads.

**Figure S12: Low-quality ASBs are not better conserved than non-ASB sites. (A)** No significant difference is found between the number of (A) highly conserved SNPs or the number of **(B)** non-conserved SNPs when compared to the non-ASB set using a counts threshold > 100. Similar results are found for a counts threshold > 50 to determine non-ASB sets in **(C)** and **(D).**

**Figure S13: Trait/QTL associations for low-quality ASBs compared to non-ASB comparator sets***.* **(A)** As these non-ASB sets were matched on minor allele frequency (MAF), we see no significant difference between the MAFs for the low-quality ASB and non-ASB sets with p-values of 0.9 and 0.9 for the count 50 and count 100 thresholds, respectively. For low-quality ASBs, we find an enrichment for the **(B)** number of associated traits, but not for the number of colocalised **(C)** eQTLs, **(D)** sQTLs or **(E)** maximum V2G score. Data is queried from OpenTargets for the median non-ASB set for counts threshold of 100 and 50 and the low-quality set. The median non-ASB set was defined by choosing the SNP with the median MAF across all 1000 sampled sets, for each SNP (Methods). Note that the y-axis in plots **(B-E)** is log10-scaled.

**Figure S14: Concordance rates in overlapping SNP/TF pairs tested across databases.** For all SNP-TF pairs in (A) ADASTRA, or (B) GVATdb, or (C) all motif-concordant SNP pairs in AlleleDb.

**Figure S15: Overlap in motif-concordant ASBs between ADASTRA and *baal-nf* ASB databases.** The ADASTRA database maps to known motifs found in the HOCOMOCO motif resource, and *baal-nf* maps to JASPAR and NoPeak motifs. For TF-SNP pairs tested in both approaches, we looked at overlap of high-quality ASBs found in both approaches. *baal-nf* can recover more high-quality ASBs than ADASTRA, despite *baal-nf* analysing fewer ChIP-seq samples (*n* = 6,925) than ADASTRA (*n =* 11,394). In part, this is due to *baal-nf* performing *de novo* motif discovery using NoPeak in the motif-mapping workflow, extending the set of biologically-relevant motifs investigated.

**Figure S16: UpSet plot showing overlapping ASB calls between GVATdb and *baal-nf***. Overlap of SNP-TF pairs that were found to be either an ASB or non-ASB in *baal-nf* and GVATdb.

**Figure S17: UpSet Plot showing overlapping motif-concordant ASB calls across AlleleDb and *baal-nf****.* Overlap of SNP-TF pairs that were found to be either an ASB or non-ASB in *baal-nf* and AlleleDb.

**Figure S18: Flow chart detailing the sampling procedure for generating non-ASB sets from a reference set** (high-quality ASB set as reference shown here). All SNP-TF-cell line combinations that were tested with *baal-nf* were broken down into various subsets – high-quality ASBs (green; top), non-ASBs with at least 50 total read counts covering that SNP (orange; middle) and non-ASBs with at least 100 total read counts covering that SNP (yellow; bottom). The high-quality set is used to match MAF within the two non-ASB sets before sampling with replacement to derive the non-ASB comparator sets shown on the right.

**Figure S19: Pipeline for evaluating evolutionary, functional and trait relevance of non-ASB sets compared to reference ASB set** (shown here as high-quality ASB set). All non-ASB comparator sets (grey) as well as the high-quality set (green) are assessed for evolutionary conservation using PhastCons as well as trait/QTL associations in OpenTargets.

Supplemental Note

*Comparison of* baal-nf *to existing ASB databases*

Here, we compare *baal-nf* results to those in other ASB databases, specifically ADASTRA(10), GTRD(49), GVATdb(51), and AlleleDB(48). ADASTRA is comparable to *baal-nf* in scale and investigated ChIP-seq datasets and so will be elaborated on in greater detail.

ADASTRA and *baal-nf*’s minimal ASB overlap is largely due to these approaches’ investigated TF-SNP pairs being largely disjoint: only 278,113 TF-SNP pairs were tested by both (Main text). When the same TF-SNP pairs were tested by both ADASTRA and *baal-nf*, however, 95.3% (264,915 of 278,113) yielded concordant results: 262,489 non-ASBs and 2,426 ASBs (Additional file 1: Figure S14A). For the remaining 13,198 (4.7%), 5,007 and 8,191 were called as ASBs only by ADASTRA or by *baal-nf*, respectively. Discordant results could result from these methods’ analyses of different ChIP-seq datasets in different cell lines [Figure 4C] with potentially different read coverage at heterozygous SNPs. They could also arise from methodological differences in CNV calls: ADASTRA uses ChIP-seq reads to both call genotypes and estimate background allelic dosage. Further, ADASTRA maps SNPs to motifs using the HOCOMOCO, rather than JASPAR, database, and does not include *de novo* motif discovery. Notably, among ASBs assigned to the same motif by the two methods, *baal-nf* discovered a larger set of high-quality ASBs (n=1,020) than ADASTRA (n=684), with 218 motif-concordant ASBs being discovered by both approaches [Additional file 1: Figure S15, Methods].

Of the 322 TFs that were investigated in both approaches, only 21 were assessed using the same ENCODE datasets and can therefore be compared directly [Figure 4D]. For these 21 TFs and their associated 25 ENCODE datasets, 14,194 SNP-TF pairs were assessed by both ADASTRA and *baal-nf,* with 102 called as an ASB by ADASTRA, 533 called as an ASB by *baal-nf*, and 50 called by both approaches. Methodological differences that could drive differences between ADASTRA and *baal-nf* relate to different (i) data pre-processing of ChIP-seq reads, (ii) CNV estimation (as above), and (iii) statistical models used to infer allele bias.

Read coverage and CNV estimation for each SNP were available from ADASTRA and could therefore be compared directly with *baal-nf*. ASBs discovered by ADASTRA, but not *baal-nf*, tended to have higher read coverage in ADASTRA compared to *baal-nf* at assessed heterozygous SNPs (p-value= 9.4 x 10^-5^) [Figure 4E]. *baal-nf* implements different read QC steps than are employed for the GTRD database used by ADASTRA, including the implementation of FastQScreen in *baal-nf*, but not ADASTRA, to filter out additional reads that could be contaminants(10). This leads to lower read coverage at some heterozygous SNPs in *baal-nf* and reduces evidence for ASB calls.

ASBs that were detected by *baal-nf,* but not ADASTRA, displayed quantitative differences in how CNVs were estimated across both approaches. In ADASTRA, CNVs are estimated using BABACHI. This builds background allelic dosage maps using ChIP-seq reads across heterozygous SNPs organised into sub-chromosomal regions. BABACHI significantly underestimates the RAF (as defined by the BAF reported in ENCODE for that same cell line) at assessed heterozygous SNPs [Figure 4F], which will impact ASB inference by correcting for CNV bias differently across the methods (p-value=4.1 x 10^-3^). This comparison is also directly relevant to GTRD because its reported ASBs are derived from ADASTRA.

Prediction incompleteness is also evident when comparing *baal-nf* ASBs to those predicted from SNP evaluation by Systematic Evolution of Ligands by EXponential enrichment (SNP-SELEX) data in GVATdb(51). Compared with GVATdb, *baal-nf* tested 17.5-fold more TF-SNP pairs and called 13.5-fold more ASBs, hence showing similar ASB call rates. Few identical TF-SNP pairs (with the same REF/ALT alleles) were tested in both strategies: 3,170 of 1,374,477 (0.2% of GVATdb), and 3,170 of 23,997,518 (0.01% of *baal-nf*). Among these 3,170 TF-SNP pairs, there were 4,435 ASB tests applied owing to multiple experiments and/or cell lines. Of these pairs, 3,083 (97.3%) were called as non-ASBs by both; 83 (2.6%) were discordant, being called as a non-ASB by *baal-nf* and as an ASB in GVATdb, or vice versa; and 4 (0.1%) were called as an ASB by both *baal-nf* and in GVATdb (Additional file 1: Figure S14B, Additional file 1: Figure S16). As discussed above, a TF-SNP pair could be called as both a non-ASB and ASB by these approaches due to experimental differences.

Finally, we compared *baal-nf* ASBs to those reported in AlleleDb(48). AlleleDb predicts ASBs using ENCODE ChIP-seq datasets, some of which are those investigated in *baal-nf* allowing us to compare TF/cell line pairs across the two databases. All 16 motif-concordant ASBs reported in AlleleDb that were tested in *baal-nf*, were called as ASBs by *baal-nf* (Additional file 1: Figure S14C). Moreover, these ASBs were also all concordant with respect to (i) binding affinity direction, and (ii) the TF binding motif used in *baal-nf*. A further 213 and 823 ASBs were tested only in AlleleDb or in *baal-nf*, respectively (Additional file 1: Figure S17). For TF/cell lines that matched across the two databases, all 134,349 non-ASBs in *baal-nf* were not called as motif-concordant ASBs by AlleleDb (Additional file 1: Figure S17). In summary, when the same TF-SNP pair is tested across methods, results are consistent.
